# Diaqua­bis­(5-carb­oxy-2-propyl-1*H*-imidazole-4-carboxyl­ato-κ^2^ *N* ^3^,*O* ^4^)cadmium(II) 3.5-hydrate

**DOI:** 10.1107/S1600536810031466

**Published:** 2010-08-28

**Authors:** Shi-Jie Li, Jing-Jing Dong, Wen-Dong Song, Jian-Bin Yan, Shi-Hong Li

**Affiliations:** a College of Food Science and Technology, Guangdong Ocean University, Zhanjiang 524088, People’s Republic of China; b College of Science, Guangdong Ocean University, Zhanjiang 524088, People’s Republic of China; c College of Medical Laboratory, Hebei North University, Zhangjiakou 075000, People’s Republic of China

## Abstract

In the title complex, [Cd(C_8_H_9_N_2_O_4_)_2_(H_2_O)_2_]·3.5H_2_O, the Cd^II^ is coordinated by two water mol­ecules and *N*,*O*-chelated by two 5-carb­oxy-2-propyl-1*H*-imidazole-4-carboxyl­ate anions in a distorted octa­hedral geometry. The two imidazole rings are oriented to each other with a dihedral angle of 75.1 (2)°. Strong O—H⋯O hydrogen bonds between protonated and deprotonated carboxyl­ate groups occur in the mol­ecular structure. In the crystal structure extensive O—H⋯O and N—H⋯O hydrogen bonds help to stabilize the three-dimensional supra­molecular framework. The propyl groups of anions are disordered over two sites with refined occupancies of 0.768 (6):0.232 (6) and 0.642 (8):0.358 (8).

## Related literature

For the potential uses and diverse structural types of metal complexes with the imidazole-4,5-dicarboxyl­ate ligand, see: Zou *et al.* (2006 ▶); Li *et al.* (2006 ▶); Liu *et al.* (2004 ▶); Sun *et al.* (2005 ▶). For related structures, see: Yan *et al.* (2010 ▶); Li *et al.* (2010 ▶); Song *et al.* (2010 ▶); He *et al.* (2010 ▶); Fan *et al.* (2010 ▶).
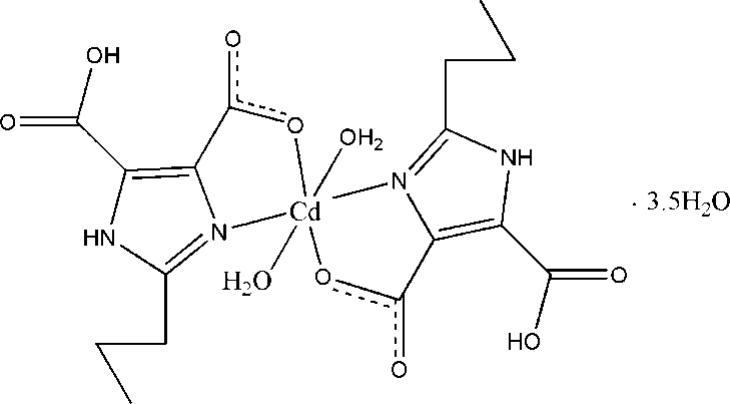


## Experimental

### 

#### Crystal data

[Cd(C_8_H_9_N_2_O_4_)_2_(H_2_O)_2_]·3.5H_2_O
*M* *_r_* = 605.83
Triclinic, 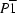

*a* = 10.6228 (12) Å
*b* = 10.7000 (12) Å
*c* = 11.3694 (13) Å
α = 83.690 (1)°
β = 81.701 (1)°
γ = 87.441 (1)°
*V* = 1270.5 (2) Å^3^
*Z* = 2
Mo *K*α radiation
μ = 0.93 mm^−1^
*T* = 296 K
0.29 × 0.24 × 0.21 mm

#### Data collection

Bruker APEXII area-detector diffractometer
Absorption correction: multi-scan (*SADABS*; Bruker, 2004 ▶) *T*_min_ = 0.775, *T*_max_ = 0.829
6589 measured reflections
4494 independent reflections
3730 reflections with *I* > 2σ(*I*)
*R*_int_ = 0.034

#### Refinement

*R*[*F* ^2^ > 2σ(*F* ^2^)] = 0.041
*wR*(*F* ^2^) = 0.107
*S* = 1.05
4494 reflections
346 parameters
7 restraints
H atoms treated by a mixture of independent and constrained refinement
Δρ_max_ = 0.46 e Å^−3^
Δρ_min_ = −0.59 e Å^−3^

### 

Data collection: *APEX2* (Bruker, 2004 ▶); cell refinement: *SAINT* (Bruker, 2004 ▶); data reduction: *SAINT*; program(s) used to solve structure: *SHELXS97* (Sheldrick, 2008 ▶); program(s) used to refine structure: *SHELXL97* (Sheldrick, 2008 ▶); molecular graphics: *SHELXTL* (Sheldrick, 2008 ▶); software used to prepare material for publication: *SHELXTL*.

## Supplementary Material

Crystal structure: contains datablocks I, global. DOI: 10.1107/S1600536810031466/xu5001sup1.cif

Structure factors: contains datablocks I. DOI: 10.1107/S1600536810031466/xu5001Isup2.hkl

Additional supplementary materials:  crystallographic information; 3D view; checkCIF report

## Figures and Tables

**Table 1:** Hydrogen-bond geometry (Å, °)

*D*—H⋯*A*	*D*—H	H⋯*A*	*D*⋯*A*	*D*—H⋯*A*
N2—H2⋯O4*W*	0.86	1.89	2.747 (5)	172
N4—H4⋯O6*W*	0.86	1.90	2.729 (5)	161
O1—H1⋯O4	0.82 (3)	1.67 (3)	2.490 (5)	172 (7)
O7—H7⋯O6	0.82 (4)	1.63 (4)	2.440 (5)	171 (8)
O1*W*—H1*W*⋯O5*W*^i^	0.85	2.27	2.654 (5)	108
O1*W*—H2*W*⋯O8^ii^	0.85	1.90	2.737 (5)	168
O2*W*—H3*W*⋯O8^iii^	0.84	2.08	2.852 (5)	152
O2*W*—H4*W*⋯O2^iv^	0.86	1.99	2.818 (5)	163
O3*W*—H5*W*⋯O3^iv^	0.85	2.19	2.794 (7)	128
O3*W*—H6*W*⋯O3^v^	0.85	1.96	2.770 (6)	160
O4*W*—H7*W*⋯O3*W*	0.85	1.78	2.620 (8)	169
O4*W*—H8*W*⋯O7^vi^	0.85	2.05	2.904 (6)	180
O5*W*—H9*W*⋯O5^vi^	0.87	2.01	2.841 (5)	159
O5*W*—H10*W*⋯O4^vii^	0.85	1.95	2.778 (5)	163
O6*W*—H11*W*⋯O3*W*^vii^	0.85	1.87	2.679 (8)	160
O6*W*—H12*W*⋯O5*W*^viii^	0.85	2.22	2.850 (5)	130

Symmetry codes: (i) 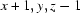; (ii) 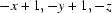; (iii) 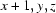; (iv) 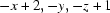; (v) 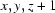; (vi) 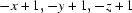; (vii) 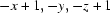; (viii) 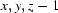.

**Fig. 1.**
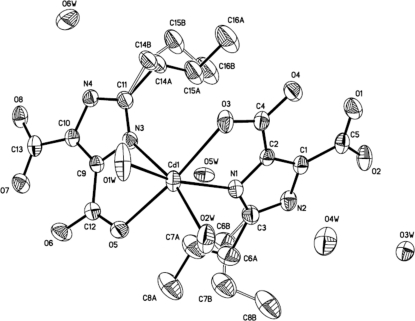
The structure of the title compound, showing the atomic-numbering scheme. Non-H atoms are shown with 30% probability displacement ellipsoids.

## supplementary crystallographic information

### Comment

Imidazole-4,5-dicarboxylic acid (H_3_IDC) has been widely used to coordinate with metal salts to obtain a series of MOFs with different structures and useful properties(Zou *et al.*, 2006; Li *et al.*, 2006; Liu *et al.*, 2004; Sun *et al.*, 2005), 2-propyl-1*H*-imidazole-4,5-carboxylate(H_3_pimda) ligand as one derivative of H_3_IDC with efficient N,*O*-donors has been used to obtain new metal-organic complexes by our research group, such as poly[diaquabis(5-carboxy-2-propyl-1*H*-imidazole-4-carboxylato- k^3^ N^3^,*O*^4^,*O*^5^)calcium(II)] (Song *et al.*, 2010), [diaquabis (5-carboxy-2-propyl-1*H*-imidazole-4-carboxylato-k^2^N^3^,*O*^4^) manganese(II)]*N*,*N*-dimethylformamide (Yan *et al.*, 2010), [Diaquabis(5-carboxy-2-propyl-1*H*-imidazole-4-carboxylato-k^2^*N*^3^,*O*^4^)nickle(II)]*N*,*N*-dimethylformamide disolvate(Li *et al.*, 2010), Diaquabis(4-carboxy-2-propyl-1*H*-imidazole-5-carboxylato- k^2^N^3^,*O*^4^)copper(II) *N*,*N*-dimethylformamide disolvate (He *et al.*, 2010) and Diaquabis(5-carboxy-2-propyl-1*H*-imidazole- 4-carboxylato-k^2^N^3^,*O*^4^)nickle(II) tetrahedrate (Fan *et al.*, 2010). In this paper, we report the structure of a new Cd(II) complex obtained under hydrothermal conditions.

As illustrated in figure 1, the title complex molecule is similar to diaquabis(5-carboxy-2-propyl-1*H*-imidazole-4-carboxylato- κ^2^*N*,*O*)nickle(II) tetrahedrate (Fan *et al.* 2010), contains one Cd^II^ ion, two mono-deprotonated H_2_pimda^-^ anions, two coordinated water molecules and 3.5 solvent water molecules. The Cd^II^ is six-coordinated by two N,*O*-bidentate H_3_pimda anions and two water molecules in a distorted octahedral geometry. the dihedral angle between the two imidazole rings is 75.0 (1) %A. In the crystal structure, the three-dimensional supramolecular framework is stabilized by extensive O—H···O and N—H···O hydrogen bonds involving the free water molecules, the coordinated water molecules, the carboxy O atoms and the protonated N atoms of H_3_pimda. The propyl groups of H_3_pimda are disordered over two sets of sites with refined occupiencies of 0.772 (6):0.228 (6) and 0.642 (8):0.358 (8).

### Experimental

A mixture of CdCl_2_ (0.5 mmol, 0.09 g) and 2-propyl-1*H*-imidazole-4,5-dicarboxylic acid (0.5 mmol, 0.99 g) in 15 ml water was sealed in an autoclave equipped with a Teflon liner (20 ml) and then heated at 433 K for 4 days. Crystals of the title compound were obtained by slow evaporation of the solvent at room temperature.

### Refinement

Water H atoms were located in a difference Fourier map and were allowed to ride on the parent atom, with *U*_iso_(H) = 1.5*U*_eq_(O). Carboxyl H atoms were located in a difference map and refined with distance restraints, *U*_iso_(H) = 1.5*U*_eq_(O). Other H atoms were placed at calculated positions and were treated as riding on parent atoms with C—H = 0.96 (methyl), 0.97 (methylene) and N—H = 0.86 Å, *U*_iso_(H) = 1.2 or 1.5*U*_eq_(C,*N*). The propyl groups of H_3_pimda are disordered over two sites with refined occupancies of 0.768 (6):0.232 (6) and 0.642 (8):0.358 (8). C—C distance restraints of disordered components were applied. The O3W water molecule is located close to an inversion center, its occupancy factor was refined to 0.49 (1) and was fixed as 0.5 at the final refinements.

### Crystal data

**Table:**

[Cd(C_8_H_9_N_2_O_4_)_2_(H_2_O)_2_]·3.5H_2_O	*Z* = 2
*M**_r_* = 605.83	*F*(000) = 618
Triclinic, *P*1	*D*_x_ = 1.584 Mg m^−^^3^
Hall symbol: -P 1	Mo *K*α radiation, λ = 0.71073 Å
*a* = 10.6228 (12) Å	Cell parameters from 3600 reflections
*b* = 10.7000 (12) Å	θ = 1.4–25.0°
*c* = 11.3694 (13) Å	µ = 0.93 mm^−^^1^
α = 83.690 (1)°	*T* = 296 K
β = 81.701 (1)°	Block, colorless
γ = 87.441 (1)°	0.29 × 0.24 × 0.21 mm
*V* = 1270.5 (2) Å^3^	

### Data collection

**Table:**

Bruker APEXII area-detector diffractometer	4494 independent reflections
Radiation source: fine-focus sealed tube	3730 reflections with *I* > 2σ(*I*)
graphite	*R*_int_ = 0.034
φ and ω scan	θ_max_ = 25.2°, θ_min_ = 1.9°
Absorption correction: multi-scan (*SADABS*; Bruker, 2004)	*h* = −12→12
*T*_min_ = 0.775, *T*_max_ = 0.829	*k* = −12→8
6589 measured reflections	*l* = −13→13

### Refinement

**Table:**

Refinement on *F*^2^	Primary atom site location: structure-invariant direct methods
Least-squares matrix: full	Secondary atom site location: difference Fourier map
*R*[*F*^2^ > 2σ(*F*^2^)] = 0.041	Hydrogen site location: inferred from neighbouring sites
*wR*(*F*^2^) = 0.107	H atoms treated by a mixture of independent and constrained refinement
*S* = 1.05	*w* = 1/[σ^2^(*F*_o_^2^) + (0.0505*P*)^2^ + 0.1685*P*] where *P* = (*F*_o_^2^ + 2*F*_c_^2^)/3
4494 reflections	(Δ/σ)_max_ = 0.001
346 parameters	Δρ_max_ = 0.46 e Å^−^^3^
7 restraints	Δρ_min_ = −0.59 e Å^−^^3^

### Special details

**Table:**

Geometry. All e.s.d.'s (except the e.s.d. in the dihedral angle between two l.s. planes) are estimated using the full covariance matrix. The cell e.s.d.'s are taken into account individually in the estimation of e.s.d.'s in distances, angles and torsion angles; correlations between e.s.d.'s in cell parameters are only used when they are defined by crystal symmetry. An approximate (isotropic) treatment of cell e.s.d.'s is used for estimating e.s.d.'s involving l.s. planes.
Refinement. Refinement of *F*^2^ against ALL reflections. The weighted *R*-factor *wR* and goodness of fit *S* are based on *F*^2^, conventional *R*-factors *R* are based on *F*, with *F* set to zero for negative *F*^2^. The threshold expression of *F*^2^ > σ(*F*^2^) is used only for calculating *R*-factors(gt) *etc*. and is not relevant to the choice of reflections for refinement. *R*-factors based on *F*^2^ are statistically about twice as large as those based on *F*, and *R*- factors based on ALL data will be even larger.

### Fractional atomic coordinates and isotropic or equivalent isotropic displacement parameters (Å^2^)

**Table:**

	*x*	*y*	*z*	*U*_iso_*/*U*_eq_	Occ. (<1)
Cd1	0.84023 (3)	0.29169 (3)	0.19976 (2)	0.04816 (13)	
O1	0.8778 (4)	−0.1913 (3)	0.5186 (3)	0.0698 (9)	
O2	0.8413 (3)	−0.1136 (3)	0.6913 (3)	0.0701 (9)	
O3	0.8810 (3)	0.0754 (3)	0.1940 (2)	0.0565 (7)	
O4	0.8944 (3)	−0.1083 (3)	0.3037 (3)	0.0656 (8)	
O5	0.7595 (3)	0.5000 (3)	0.2111 (3)	0.0553 (7)	
O6	0.5964 (3)	0.6304 (3)	0.1835 (3)	0.0660 (8)	
O7	0.3809 (3)	0.6208 (3)	0.1350 (3)	0.0604 (8)	
O8	0.2516 (3)	0.4784 (3)	0.0948 (3)	0.0606 (8)	
N1	0.8262 (3)	0.2015 (3)	0.3933 (3)	0.0454 (7)	
N2	0.8116 (3)	0.1310 (3)	0.5823 (3)	0.0550 (9)	
H2	0.8009	0.1302	0.6588	0.066*	
N3	0.6294 (3)	0.2956 (3)	0.1734 (3)	0.0438 (7)	
N4	0.4358 (3)	0.2862 (3)	0.1287 (3)	0.0479 (8)	
H4	0.3681	0.2556	0.1112	0.058*	
C1	0.8362 (3)	0.0280 (4)	0.5209 (3)	0.0447 (9)	
C2	0.8454 (3)	0.0742 (3)	0.4021 (3)	0.0395 (8)	
C3	0.8069 (5)	0.2337 (4)	0.5040 (4)	0.0591 (11)	
C4	0.8760 (4)	0.0090 (4)	0.2913 (3)	0.0473 (9)	
C5	0.8512 (4)	−0.0992 (4)	0.5834 (4)	0.0517 (10)	
C6A	0.7979 (9)	0.3683 (10)	0.5325 (10)	0.085 (3)	0.772 (6)
H6A	0.8215	0.3717	0.6114	0.102*	0.772 (6)
H6B	0.8579	0.4174	0.4753	0.102*	0.772 (6)
C7A	0.6674 (9)	0.4249 (7)	0.5292 (8)	0.107 (3)	0.772 (6)
H7A	0.6091	0.3854	0.5949	0.128*	0.772 (6)
H7B	0.6379	0.4108	0.4550	0.128*	0.772 (6)
C8A	0.6699 (11)	0.5651 (7)	0.5390 (9)	0.129 (4)	0.772 (6)
H8A	0.5848	0.5970	0.5604	0.193*	0.772 (6)
H8B	0.7059	0.6073	0.4635	0.193*	0.772 (6)
H8C	0.7207	0.5796	0.5992	0.193*	0.772 (6)
C6B	0.740 (4)	0.350 (4)	0.549 (4)	0.085 (3)	0.228 (6)
H6C	0.6751	0.3816	0.5002	0.102*	0.228 (6)
H6D	0.6998	0.3328	0.6313	0.102*	0.228 (6)
C7B	0.844 (3)	0.442 (3)	0.540 (3)	0.107 (3)	0.228 (6)
H7C	0.8078	0.5259	0.5500	0.128*	0.228 (6)
H7D	0.8951	0.4445	0.4616	0.128*	0.228 (6)
C8B	0.924 (3)	0.399 (3)	0.637 (3)	0.129 (4)	0.228 (6)
H8D	0.9722	0.4679	0.6515	0.193*	0.228 (6)
H8E	0.9808	0.3317	0.6117	0.193*	0.228 (6)
H8F	0.8695	0.3699	0.7086	0.193*	0.228 (6)
C9	0.5736 (3)	0.4138 (3)	0.1688 (3)	0.0397 (8)	
C10	0.4529 (3)	0.4099 (4)	0.1405 (3)	0.0413 (8)	
C11	0.5431 (4)	0.2209 (4)	0.1490 (4)	0.0509 (10)	
C12	0.6485 (4)	0.5201 (4)	0.1898 (3)	0.0470 (9)	
C13	0.3529 (4)	0.5090 (4)	0.1221 (3)	0.0481 (9)	
C14A	0.5509 (17)	0.0797 (5)	0.1655 (9)	0.061 (3)	0.642 (8)
H14A	0.6361	0.0508	0.1353	0.073*	0.642 (8)
H14B	0.4918	0.0464	0.1201	0.073*	0.642 (8)
C15A	0.5189 (11)	0.0302 (8)	0.2979 (9)	0.081 (3)	0.642 (8)
H15A	0.5740	0.0680	0.3443	0.098*	0.642 (8)
H15B	0.4315	0.0537	0.3267	0.098*	0.642 (8)
C16A	0.5361 (10)	−0.1097 (8)	0.3138 (11)	0.108 (4)	0.642 (8)
H16A	0.5605	−0.1354	0.3912	0.161*	0.642 (8)
H16B	0.6013	−0.1358	0.2531	0.161*	0.642 (8)
H16C	0.4576	−0.1479	0.3075	0.161*	0.642 (8)
C14B	0.572 (3)	0.0878 (9)	0.119 (2)	0.061 (3)	0.358 (8)
H14C	0.6611	0.0664	0.1216	0.073*	0.358 (8)
H14D	0.5526	0.0790	0.0400	0.073*	0.358 (8)
C15B	0.4893 (14)	0.0002 (15)	0.2123 (14)	0.081 (3)	0.358 (8)
H15C	0.4005	0.0244	0.2101	0.098*	0.358 (8)
H15D	0.5017	−0.0851	0.1905	0.098*	0.358 (8)
C16B	0.518 (3)	0.002 (2)	0.3363 (17)	0.108 (4)	0.358 (8)
H16D	0.4796	−0.0688	0.3859	0.161*	0.358 (8)
H16E	0.4832	0.0784	0.3669	0.161*	0.358 (8)
H16F	0.6080	−0.0025	0.3363	0.161*	0.358 (8)
O1W	0.8927 (3)	0.3292 (4)	0.0015 (3)	0.0955 (13)	
H1W	0.9095	0.2617	−0.0314	0.143*	
H2W	0.8569	0.3905	−0.0363	0.143*	
O2W	1.0419 (3)	0.3400 (4)	0.2218 (3)	0.0897 (12)	
H3W	1.0847	0.4010	0.1869	0.135*	
H4W	1.0911	0.2778	0.2405	0.135*	
O3W	0.9149 (6)	0.0145 (6)	0.9607 (5)	0.0625 (15)	0.50
H5W	0.9951	0.0020	0.9528	0.094*	0.50
H6W	0.9090	0.0145	1.0360	0.094*	0.50
O4W	0.7737 (5)	0.1534 (4)	0.8233 (3)	0.1211 (17)	
H7W	0.8269	0.1083	0.8598	0.182*	
H8W	0.7288	0.2197	0.8353	0.182*	
O5W	0.1097 (3)	0.2735 (3)	0.8678 (3)	0.0718 (9)	
H9W	0.1608	0.3312	0.8299	0.108*	
H10W	0.1151	0.2123	0.8255	0.108*	
O6W	0.2598 (4)	0.1582 (4)	0.0406 (4)	0.1044 (13)	
H11W	0.2130	0.0949	0.0534	0.157*	
H12W	0.2045	0.2161	0.0283	0.157*	
H1	0.876 (8)	−0.162 (7)	0.449 (2)	0.157*	
H7	0.453 (3)	0.632 (7)	0.149 (7)	0.157*	

### Atomic displacement parameters (Å^2^)

**Table:**

	*U*^11^	*U*^22^	*U*^33^	*U*^12^	*U*^13^	*U*^23^
Cd1	0.04170 (18)	0.0518 (2)	0.0479 (2)	0.00347 (13)	−0.00891 (12)	0.01025 (13)
O1	0.091 (2)	0.0495 (19)	0.067 (2)	−0.0007 (16)	−0.0173 (19)	0.0084 (16)
O2	0.070 (2)	0.079 (2)	0.0510 (19)	0.0116 (17)	−0.0047 (15)	0.0254 (15)
O3	0.0718 (19)	0.0587 (18)	0.0362 (15)	0.0191 (14)	−0.0077 (13)	−0.0008 (13)
O4	0.096 (2)	0.0453 (18)	0.0579 (18)	0.0127 (16)	−0.0199 (16)	−0.0092 (14)
O5	0.0471 (16)	0.0495 (17)	0.0723 (19)	−0.0052 (13)	−0.0187 (14)	−0.0049 (14)
O6	0.0647 (19)	0.0398 (17)	0.096 (2)	0.0045 (14)	−0.0164 (17)	−0.0127 (16)
O7	0.0518 (17)	0.0510 (18)	0.077 (2)	0.0153 (14)	−0.0098 (15)	−0.0072 (15)
O8	0.0370 (15)	0.069 (2)	0.071 (2)	0.0022 (13)	−0.0108 (13)	0.0172 (15)
N1	0.0490 (18)	0.0434 (19)	0.0429 (18)	0.0051 (14)	−0.0080 (14)	−0.0005 (14)
N2	0.063 (2)	0.064 (2)	0.0355 (17)	0.0098 (17)	−0.0058 (15)	−0.0041 (16)
N3	0.0387 (17)	0.0403 (18)	0.0517 (19)	0.0014 (14)	−0.0099 (14)	0.0012 (14)
N4	0.0384 (17)	0.050 (2)	0.055 (2)	−0.0057 (14)	−0.0133 (14)	0.0032 (15)
C1	0.038 (2)	0.049 (2)	0.044 (2)	0.0000 (16)	−0.0035 (16)	0.0043 (18)
C2	0.0334 (18)	0.044 (2)	0.041 (2)	0.0010 (15)	−0.0074 (15)	−0.0007 (16)
C3	0.076 (3)	0.053 (3)	0.050 (3)	0.009 (2)	−0.014 (2)	−0.006 (2)
C4	0.047 (2)	0.050 (2)	0.045 (2)	0.0074 (17)	−0.0120 (17)	−0.0020 (18)
C5	0.043 (2)	0.061 (3)	0.047 (2)	0.0004 (18)	−0.0083 (18)	0.014 (2)
C6A	0.109 (9)	0.068 (5)	0.078 (5)	0.011 (6)	−0.009 (6)	−0.021 (4)
C7A	0.133 (8)	0.069 (5)	0.115 (7)	0.011 (5)	−0.004 (5)	−0.017 (5)
C8A	0.174 (10)	0.067 (5)	0.136 (8)	0.020 (5)	0.006 (7)	−0.012 (5)
C6B	0.109 (9)	0.068 (5)	0.078 (5)	0.011 (6)	−0.009 (6)	−0.021 (4)
C7B	0.133 (8)	0.069 (5)	0.115 (7)	0.011 (5)	−0.004 (5)	−0.017 (5)
C8B	0.174 (10)	0.067 (5)	0.136 (8)	0.020 (5)	0.006 (7)	−0.012 (5)
C9	0.0391 (19)	0.039 (2)	0.0383 (19)	0.0003 (15)	−0.0029 (15)	0.0024 (15)
C10	0.039 (2)	0.044 (2)	0.0378 (19)	−0.0004 (16)	0.0002 (15)	0.0032 (16)
C11	0.048 (2)	0.043 (2)	0.061 (3)	−0.0025 (18)	−0.0127 (19)	0.0029 (19)
C12	0.048 (2)	0.046 (2)	0.047 (2)	0.0007 (18)	−0.0077 (17)	−0.0012 (17)
C13	0.041 (2)	0.056 (3)	0.040 (2)	0.0088 (18)	0.0033 (16)	0.0075 (18)
C14A	0.061 (7)	0.036 (3)	0.087 (9)	−0.006 (3)	−0.022 (7)	0.007 (4)
C15A	0.062 (5)	0.058 (5)	0.121 (8)	−0.003 (4)	−0.028 (5)	0.023 (5)
C16A	0.101 (7)	0.059 (5)	0.157 (10)	−0.007 (5)	−0.023 (6)	0.023 (6)
C14B	0.061 (7)	0.036 (3)	0.087 (9)	−0.006 (3)	−0.022 (7)	0.007 (4)
C15B	0.062 (5)	0.058 (5)	0.121 (8)	−0.003 (4)	−0.028 (5)	0.023 (5)
C16B	0.101 (7)	0.059 (5)	0.157 (10)	−0.007 (5)	−0.023 (6)	0.023 (6)
O1W	0.085 (3)	0.121 (3)	0.059 (2)	0.052 (2)	0.0107 (18)	0.035 (2)
O2W	0.0499 (19)	0.100 (3)	0.110 (3)	−0.0224 (18)	−0.0251 (18)	0.057 (2)
O3W	0.065 (4)	0.075 (4)	0.048 (3)	0.008 (3)	−0.007 (3)	−0.013 (3)
O4W	0.156 (4)	0.142 (4)	0.072 (2)	0.058 (3)	−0.030 (3)	−0.048 (3)
O5W	0.0623 (19)	0.069 (2)	0.084 (2)	−0.0175 (16)	0.0083 (16)	−0.0255 (17)
O6W	0.101 (3)	0.098 (3)	0.131 (4)	−0.001 (2)	−0.064 (3)	−0.025 (3)

### Geometric parameters (Å, °)

**Table:**

Cd1—O1W	2.238 (3)	C6B—H6D	0.9700
Cd1—O2W	2.281 (3)	C7B—C8B	1.512 (14)
Cd1—N1	2.290 (3)	C7B—H7C	0.9700
Cd1—N3	2.300 (3)	C7B—H7D	0.9700
Cd1—O3	2.341 (3)	C8B—H8D	0.9600
Cd1—O5	2.362 (3)	C8B—H8E	0.9600
O1—C5	1.291 (5)	C8B—H8F	0.9600
O1—H1	0.82 (3)	C9—C10	1.370 (5)
O2—C5	1.209 (5)	C9—C12	1.476 (5)
O3—C4	1.243 (5)	C10—C13	1.487 (5)
O4—C4	1.257 (5)	C11—C14A	1.502 (7)
O5—C12	1.243 (5)	C11—C14B	1.507 (9)
O6—C12	1.280 (5)	C14A—C15A	1.534 (12)
O7—C13	1.275 (5)	C14A—H14A	0.9700
O7—H7	0.82 (4)	C14A—H14B	0.9700
O8—C13	1.229 (5)	C15A—C16A	1.495 (10)
N1—C3	1.327 (5)	C15A—H15A	0.9700
N1—C2	1.363 (5)	C15A—H15B	0.9700
N2—C3	1.341 (5)	C16A—H16A	0.9600
N2—C1	1.364 (5)	C16A—H16B	0.9600
N2—H2	0.8600	C16A—H16C	0.9600
N3—C11	1.320 (5)	C14B—C15B	1.532 (14)
N3—C9	1.371 (5)	C14B—H14C	0.9700
N4—C11	1.346 (5)	C14B—H14D	0.9700
N4—C10	1.368 (5)	C15B—C16B	1.486 (13)
N4—H4	0.8600	C15B—H15C	0.9700
C1—C2	1.377 (5)	C15B—H15D	0.9700
C1—C5	1.478 (6)	C16B—H16D	0.9600
C2—C4	1.495 (5)	C16B—H16E	0.9600
C3—C6A	1.506 (11)	C16B—H16F	0.9600
C3—C6B	1.51 (4)	O1W—H1W	0.8499
C6A—C7A	1.492 (12)	O1W—H2W	0.8500
C6A—H6A	0.9700	O2W—H3W	0.8405
C6A—H6B	0.9700	O2W—H4W	0.8547
C7A—C8A	1.518 (10)	O3W—H5W	0.8500
C7A—H7A	0.9700	O3W—H6W	0.8501
C7A—H7B	0.9700	O4W—H7W	0.8503
C8A—H8A	0.9600	O4W—H8W	0.8498
C8A—H8B	0.9600	O5W—H9W	0.8741
C8A—H8C	0.9600	O5W—H10W	0.8500
C6B—C7B	1.498 (15)	O6W—H11W	0.8452
C6B—H6C	0.9700	O6W—H12W	0.8490
			
O1W—Cd1—O2W	88.94 (14)	C8B—C7B—H7C	110.3
O1W—Cd1—N1	162.70 (12)	C6B—C7B—H7D	110.3
O2W—Cd1—N1	85.50 (12)	C8B—C7B—H7D	110.3
O1W—Cd1—N3	89.03 (13)	H7C—C7B—H7D	108.5
O2W—Cd1—N3	165.95 (12)	C7B—C8B—H8D	109.5
N1—Cd1—N3	100.21 (11)	C7B—C8B—H8E	109.5
O1W—Cd1—O3	91.83 (12)	H8D—C8B—H8E	109.5
O2W—Cd1—O3	96.11 (12)	C7B—C8B—H8F	109.5
N1—Cd1—O3	72.55 (10)	H8D—C8B—H8F	109.5
N3—Cd1—O3	97.85 (10)	H8E—C8B—H8F	109.5
O1W—Cd1—O5	90.99 (12)	C10—C9—N3	110.1 (3)
O2W—Cd1—O5	94.05 (12)	C10—C9—C12	131.2 (3)
N1—Cd1—O5	105.72 (10)	N3—C9—C12	118.6 (3)
N3—Cd1—O5	72.08 (10)	N4—C10—C9	105.1 (3)
O3—Cd1—O5	169.50 (10)	N4—C10—C13	122.2 (3)
C5—O1—H1	107 (6)	C9—C10—C13	132.6 (4)
C4—O3—Cd1	117.3 (2)	N3—C11—N4	111.0 (3)
C12—O5—Cd1	115.9 (2)	N3—C11—C14A	125.1 (8)
C13—O7—H7	118 (6)	N4—C11—C14A	122.8 (8)
C3—N1—C2	106.7 (3)	N3—C11—C14B	123.8 (15)
C3—N1—Cd1	140.1 (3)	N4—C11—C14B	123.8 (16)
C2—N1—Cd1	113.2 (2)	O5—C12—O6	122.5 (4)
C3—N2—C1	108.9 (3)	O5—C12—C9	119.3 (3)
C3—N2—H2	125.6	O6—C12—C9	118.2 (4)
C1—N2—H2	125.6	O8—C13—O7	125.1 (4)
C11—N3—C9	105.6 (3)	O8—C13—C10	118.7 (4)
C11—N3—Cd1	140.3 (3)	O7—C13—C10	116.2 (4)
C9—N3—Cd1	113.7 (2)	C11—C14A—C15A	110.9 (6)
C11—N4—C10	108.1 (3)	C11—C14A—H14A	109.5
C11—N4—H4	125.9	C15A—C14A—H14A	109.5
C10—N4—H4	125.9	C11—C14A—H14B	109.5
N2—C1—C2	105.1 (3)	C15A—C14A—H14B	109.5
N2—C1—C5	121.4 (4)	H14A—C14A—H14B	108.0
C2—C1—C5	133.4 (4)	C16A—C15A—C14A	110.3 (8)
N1—C2—C1	109.3 (3)	C16A—C15A—H15A	109.6
N1—C2—C4	119.8 (3)	C14A—C15A—H15A	109.6
C1—C2—C4	130.8 (4)	C16A—C15A—H15B	109.6
N1—C3—N2	110.0 (4)	C14A—C15A—H15B	109.6
N1—C3—C6A	123.2 (6)	H15A—C15A—H15B	108.1
N2—C3—C6A	126.4 (6)	C11—C14B—C15B	108.1 (13)
N1—C3—C6B	129 (2)	C11—C14B—H14C	110.1
N2—C3—C6B	117.6 (18)	C15B—C14B—H14C	110.1
O3—C4—O4	125.2 (4)	C11—C14B—H14D	110.1
O3—C4—C2	117.2 (4)	C15B—C14B—H14D	110.1
O4—C4—C2	117.6 (3)	H14C—C14B—H14D	108.4
O2—C5—O1	122.4 (4)	C16B—C15B—C14B	113.8 (19)
O2—C5—C1	119.9 (4)	C16B—C15B—H15C	108.8
O1—C5—C1	117.6 (4)	C14B—C15B—H15C	108.8
C7A—C6A—C3	112.3 (8)	C16B—C15B—H15D	108.8
C7A—C6A—H6A	109.2	C14B—C15B—H15D	108.8
C3—C6A—H6A	109.2	H15C—C15B—H15D	107.7
C7A—C6A—H6B	109.2	C15B—C16B—H16D	109.5
C3—C6A—H6B	109.2	C15B—C16B—H16E	109.5
H6A—C6A—H6B	107.9	H16D—C16B—H16E	109.5
C6A—C7A—C8A	109.4 (8)	C15B—C16B—H16F	109.5
C6A—C7A—H7A	109.8	H16D—C16B—H16F	109.5
C8A—C7A—H7A	109.8	H16E—C16B—H16F	109.5
C6A—C7A—H7B	109.8	Cd1—O1W—H1W	112.0
C8A—C7A—H7B	109.8	Cd1—O1W—H2W	119.5
H7A—C7A—H7B	108.2	H1W—O1W—H2W	118.7
C7B—C6B—C3	104 (3)	Cd1—O2W—H3W	127.7
C7B—C6B—H6C	111.0	Cd1—O2W—H4W	116.0
C3—C6B—H6C	111.0	H3W—O2W—H4W	110.5
C7B—C6B—H6D	111.0	H5W—O3W—H6W	92.8
C3—C6B—H6D	111.0	H7W—O4W—H8W	136.0
H6C—C6B—H6D	109.0	H9W—O5W—H10W	107.5
C6B—C7B—C8B	107 (3)	H11W—O6W—H12W	100.2
C6B—C7B—H7C	110.3		

### Hydrogen-bond geometry (Å, °)

**Table:**

*D*—H···*A*	*D*—H	H···*A*	*D*···*A*	*D*—H···*A*
N2—H2···O4W	0.86	1.89	2.747 (5)	172
N4—H4···O6W	0.86	1.90	2.729 (5)	161
O1—H1···O4	0.82 (3)	1.67 (3)	2.490 (5)	172 (7)
O7—H7···O6	0.82 (4)	1.63 (4)	2.440 (5)	171 (8)
O1W—H1W···O5W^i^	0.85	2.27	2.654 (5)	108
O1W—H2W···O8^ii^	0.85	1.90	2.737 (5)	168
O2W—H3W···O8^iii^	0.84	2.08	2.852 (5)	152
O2W—H4W···O2^iv^	0.86	1.99	2.818 (5)	163
O3W—H5W···O3^iv^	0.85	2.19	2.794 (7)	128
O3W—H6W···O3^v^	0.85	1.96	2.770 (6)	160
O4W—H7W···O3W	0.85	1.78	2.620 (8)	169
O4W—H8W···O7^vi^	0.85	2.05	2.904 (6)	180
O5W—H9W···O5^vi^	0.87	2.01	2.841 (5)	159
O5W—H10W···O4^vii^	0.85	1.95	2.778 (5)	163
O6W—H11W···O3W^vii^	0.85	1.87	2.679 (8)	160
O6W—H12W···O5W^viii^	0.85	2.22	2.850 (5)	130

Symmetry codes: (i) *x*+1, *y*, *z*−1; (ii) −*x*+1, −*y*+1, −*z*; (iii) *x*+1, *y*, *z*; (iv) −*x*+2, −*y*, −*z*+1; (v) *x*, *y*, *z*+1; (vi) −*x*+1, −*y*+1, −*z*+1; (vii) −*x*+1, −*y*, −*z*+1; (viii) *x*, *y*, *z*−1.
